# Regulation of Sacha Inchi protein on fecal metabolism and intestinal microorganisms in mice

**DOI:** 10.3389/fnut.2024.1354486

**Published:** 2024-03-08

**Authors:** Kuan Wu, Wanying Gong, Shiyang Lin, Si Huang, Hongyu Mu, Mingming Wang, Jun Sheng, Cunchao Zhao

**Affiliations:** ^1^College of Food Science and Technology, Yunnan Agricultural University, Kunming, China; ^2^Pu'er Institute of Pu-erh Tea, Pu-er, China; ^3^Pu'er Agricultural Science Research Institute, Pu-er, China; ^4^Yunnan Plateau Characteristic Agricultural Industry Research Institute, Kunming, Yunnan, China; ^5^Yunnan Province Characteristic Resource Food Biological Manufacturing Engineering Research Center, Kunming, Yunnan, China

**Keywords:** Sacha Inchi protein, high-quality protein, amino acid composition, fecal metabolism, gut microbiota

## Abstract

**Introduction:**

With the increasing demand for protein utilization, exploring new protein resources has become a research hotspot. Sacha Inchi Protein (SIP) is a high-quality plant protein extracted from Sacha Inchi meal. This study aimed to investigate the impact of SIP on mouse metabolomics and gut microbiota diversity and explore the underlying pathways responsible for its health benefits.

**Methods:**

In this study, the structural composition of SIP was investigated, and the effects of SIP on fecal metabolomics and intestinal microorganisms in mice were explored by LC–MS metabolomics technology analysis and 16S rRNA gene sequencing.

**Results:**

The results showed that SIP was rich in amino acids, with the highest Manuscript Click here to view linked References content of arginine, which accounted for 22.98% of the total amino acid content; the potential fecal metabolites of mice in the SIP group involved lipid metabolism, sphingolipid metabolism, arginine biosynthesis, and amino acid metabolism; SIP altered the microbial composition of the cecum in mice, decreased the *Firmicutes*/*Bacteroidetes* value, and It decreased the abundance of the harmful intestinal bacteria *Actinobacteriota* and *Desulfobacterota*, and increased the abundance of the beneficial intestinal bacteria *Faecalibaculum, Dubosiella*.

**Discussion:**

In conclusion, SIP is a high-quality plant protein with great potential for development in lipid-lowering, intestinal health, and mental illness, providing valuable clues for further research on its health-promoting mechanisms.

## 1 Introduction

With global economic growth and population expansion, the demand and utilization of protein have been continuously increasing (1). Proteins are classified into animal protein and non-animal protein based on their sources. Animal protein mainly comes from milk, eggs, meat, and seafood, while non-animal protein primarily comes from a variety of plants such as legumes, grains, and nuts, as well as fungi, bacteria, and algae (2). Due to issues like allergenicity (3), environmental concerns (4), ethical considerations (5), and increased risk of cardiovascular diseases, metabolic syndrome, and certain cancers (6–8), people have become more thoughtful about the sources of protein intake. Research has demonstrated that plant protein plays a role in promoting health in terms of obesity, appetite, cardiovascular diseases, and muscle health (9–11). Therefore, the search for high-quality plant protein as a substitute for animal protein has become a current research focus.

Sacha Inchi (Plukenetia volubilis L.) is a plant of the Euphorbiaceae family widely cultivated in Central and South America. It is known as Sacha peanut or Inca nut (12, 13). Sacha Inchi is rich in nutrients and functionally active constituents with high economic and medicinal value and is now being promoted in other parts of the world (14).

Its seeds are considered to be a rich source of oil (35–60%), protein (25–30%), bioactive compounds, and bioactive substances including polyphenols, phytosterols, and tocopherols (15). Additionally, the seeds also contain a significant amount of essential amino acids such as cysteine, tyrosine, methionine, and tryptophan (16, 17). Sacha Inchi is recognized as a superior bioresource, and its bioactivity has been reported in various aspects, including antioxidant, anti-hypertensive, anti-cancer, cardioprotective, and immune-regulatory activities (18–22). Sacha Inchi seed is mostly used for oil extraction, and the meal is a by-product of oil extraction, which contains a large number of high-quality proteins ranging from 27 to 59.1% but is usually neglected, and the extraction and utilization of these high-quality proteins is an effective solution to the wastage of protein resources (17, 23).

The diversity and dynamics of the intestinal flora play a crucial role in the health and nutrition of the host. Intestinal flora has a profound influence on the physiological status of the host, including nutrient absorption, metabolism, and immunity, and is one of the key mediators in the maintenance of the body’s health (24). Deviations in intestinal flora have been associated with many diseases including obesity, type 2 diabetes, hepatic steatosis, intestinal bowel diseases (IBDs), and several cancers (25). Metabolites produced by gut microbes are key mediators of diet-induced host-microbe crosstalk and are relevant to host health. For example, tryptophan catabolic metabolites produced by the gut microbiota contribute to intestinal and systemic homeostasis. Microbiota-derived indole metabolites promote human and murine intestinal homeostasis through the regulation of interleukin-10 Receptor (26, 27). One study evaluated the effect of proteins on intestinal flora and found that soy proteins can regulate the composition and function of intestinal flora in animals (28). Therefore, it is important to understand the role of SIP in the regulation of intestinal flora. Metabolomics is the sum of all metabolites with molecular weights less than 1,000 Da in a biological system affected by endogenous or exogenous stimuli, including diet, disease, and treatment (29). Orally ingested compounds undergo a series of biochemical actions in the organism by the digestive system and intestinal flora. They are eventually broken down into low molecular weight metabolites, which are detected in the feces or urine by metabolomics (30). These metabolites may contribute to intestinal and systemic homeostasis in health and disease; therefore, exploring metabolic mechanisms is also necessary.

In this study, the structural composition of SIP was analyzed using amino acid analysis, Fourier-transform infrared spectroscopy (FT-IR), and scanning electron microscopy (SEM). Furthermore, LC–MS metabolomics analysis and 16S rRNA gene sequencing were employed to explore the effects of SIP on mouse fecal metabolomics and gut microbiota. The study aims to investigate the potential health mechanisms of SIP in the body, providing theoretical support for the development and utilization of SIP resources.

## 2 Materials and methods

### 2.1 Materials and chemicals

Sacha Inchi meal is provided by Xishuangbanna Inchi Bioresources Development Co., Ltd. (China, Yunnan); Whey Protein Isolate is provided by Zhengzhou Sumu Yunling Biotechnology Co., Ltd. (China, Henan); all chemical reagents used in the experiment are of analytical grade.

### 2.2 Extraction of Sacha Inchi protein

Take an appropriate amount of good color and non-moldy defatted Sacha Inchi meal. Add it to a 0.14 mol/L NaCl solution in a liquid-to-material ratio of 1:15 and mix well. Then adjust the pH to 10 with a 1 mol/L NaOH solution. Perform ultrasonic treatment at 43°C and 100 W for 15 min. Centrifuge (4,500 r/min, 10 min), take the supernatant, and adjust the pH to 4.9 with a 1 mol/L citric acid solution. Centrifuge under the same conditions, wash the precipitate with water until neutral and spray dry to obtain separated Sacha Inchi protein.

### 2.3 Structural analysis of Sacha Inchi protein

To understand the basic structure of SIP, this study employed a fully automated amino acid analyzer (Biochrom30+ from the United Kingdom) to determine the amino acid composition of SIP. The particle size and zeta (ζ) potential of SIP were measured using a zeta potential analyzer (ZS90 from Malvern Instruments Ltd., United Kingdom) equipped with a 633 nm red laser. The structure of SIP was further analyzed through Fourier transform-infrared spectroscopy (FT-IR) and scanning electron microscopy (SEM).

### 2.4 Animals and experimental design

Twenty-four 5-week-old male C57Bl/6 J mice were purchased from Henan Scbees Biotechnology Co., Ltd. (License number: SCXK 2020–0005). Before the experiment, the mice were randomly divided into 3 cages (*n* = 8). They were allowed to freely feed on a standard laboratory diet and deionized water for 7 days. All mice were housed in a controlled environment at a temperature of 25 ± 1°C with a 12-h light/12-h dark cycle and provided *ad libitum* access to food and water. After a 7-day acclimatization period, the experimental groups were administered by gavage using the appropriate test substances: control group (CON, 95% saline), Sacha Inchi Protein group (SIP, 250 mg/kg·BW), and Whey Protein Isolate group (WPI, 250 mg/kg·BW). Throughout the following 42 days, all mice had free access to food and water. On the 42nd day, the mice were sacrificed, and the cecum contents were collected under sterile conditions, rapidly frozen in liquid nitrogen, and stored at −80°C. Collect mouse feces the day before execution, hold the tail of the mouse with one hand, lift it out of the cage, collect fresh feces, place the tube of newly collected feces on ice, and then transfer to the refrigerator at −80°C for storage. The animal experiments in this study were approved by the Animal Care Committee of the College of Animal Science and Technology, Yunnan Agricultural University (Ethical Review No. 202209012).

### 2.5 Fecal metabolomics

Upon thawing, the stored samples were extracted for metabolite analysis using LC–MS. Precise amounts of samples were mixed with 400 μL of cold methanol solution (methanol: water = 4:1) and disrupted using a high-throughput tissue homogenizer at low temperature. After vortex mixing, the samples were subjected to three rounds of ice-cold ultrasonic extraction for 10 min each. The samples were then allowed to rest at −20°C for 30 min before centrifugation (13,000 g, 4°C, 15 min). Following centrifugation, the supernatant was collected and transferred to the injection vials of the LC–MS system.

LC–MS analysis was performed using the AB SCIEX UPLC-TripleTOF system. The chromatographic separation was achieved using a BEH C18 column (100 mm × 2.1 mm i.d., 1.7 μm; Waters, Milford, USA) with a mobile phase consisting of water (containing 0.1% formic acid) as solvent A and acetonitrile/isopropanol (1:1) (containing 0.1% formic acid) as solvent B. The gradient elution program was as follows: 0–3 min, 0–20% B; 3–9 min, 20–60% B; 9–11 min, 60–100% B; 11–13.5 min, 100% B; 13.5–13.6 min, 100–0% B; 13.6–16 min, 0% B. The flow rate was set at 0.40 mL/min, and the column temperature was maintained at 40°C. The samples were analyzed in both positive and negative ionization modes using electrospray ionization. The capillary voltage, cone voltage, and collision energy were set at 1.0 kV, 40 V, and 6 eV, respectively. The source temperature and desolvation temperature were set at 120°C and 500°C, respectively. The gas flow rate was 900 L/h, and the mass range for the mass spectrometer was set at m/z 50–1,000 with a resolution of 30,000. A quality control (QC) sample was prepared to assess the stability of the analytical system during the analysis.

Before statistical analysis, the raw data underwent a series of preprocessing steps. The raw data were imported into the Progenesis QI metabolomics software (Waters Corporation, Milford, USA) for baseline filtering, peak identification, integration, retention time correction, peak alignment, and data preprocessing to generate the final data matrix for subsequent analysis. The main databases utilized were http://www.hmdb.ca/ and https://metlin.scripps.edu/, along with custom-built databases. Multivariate statistical analysis was performed using SIMCA-P + 14.0 software (Umetrics, Umeå, Sweden). Python and KEGG Compound (Release 2017-05-01) were used for data analysis. The untargeted metabolomics profiling was provided by the Majorbio Cloud Platform (Shanghai, China).

### 2.6 DNA extraction, 16S rRNA gene amplification, and sequencing of cecum contents

The thawed cecum contents were subjected to 16S rRNA gene sequencing analysis. Genomic DNA was extracted from the cecum contents using the MoBio Laboratories’ fecal DNA isolation kit (Carlsbad, CA, USA). After DNA extraction, the extracted genomic DNA was validated using 1% agarose gel electrophoresis. The V3-V4 regions of the 16S rRNA gene were amplified using the ABI GeneAmp® 9,700 PCR system, and the PCR products were recovered using the AxyPrepDNA gel recovery kit (AXYGEN). The quantification of PCR products was performed using the QuantiFluor™-ST blue fluorescence quantitative system (Promega). The primers used for amplification were 338F (ACTCCTACGGGAGGCAGCAG) and 806R (GGACTACHVGGGTWTCTAAT). Following the manufacturer’s recommendations, the sequencing library was generated using the TruSeq DNA PCR-Free Sample Preparation Kit (Illumina, USA). Sequencing was performed by Majorbio (Shanghai, China) using the Illumina sequencing platform. After sequencing, the pair-end sequences were assembled using FLASH (v1.2.11), and Qiime (v1.9.1) was used for data processing. The Uparse (11) algorithm was employed for OTU clustering, and the RDP classifier (2.13) was used for sequence classification annotation (31). The data were visualized using Circos-0.67-7 to analyze the correspondence between samples and species.^1^ The microbial diversity and differential metabolites were analyzed using R and pheatmap. The above bioinformatics analyses were performed on the Majorbio Cloud Platform.

### 2.7 Data analysis

Statistical analysis was conducted using SPSS 26.0 software. One-way analysis of variance (ANOVA) and Duncan’s multiple range test were used to determine the significance of intergroup differences. Spearman correlation analysis was performed to investigate the correlation between gut microbiota composition and fecal metabolites. A value of *p* of less than 0.05 was considered statistically significant.

## 3 Results and discussion

### 3.1 Amino acid composition

In this study, the amino acid composition of SIP was analyzed. As shown in Table 1, 17 kinds of amino acids were detected in the hydrolyzed protein of SIP. Among them, 7 are Essential amino acids EAA (Essential amino acid), accounting for 26.63% of the total amino acid content, and other non-essential amino acids NEAA (Non-Essential amino acid) are also very rich. In addition, the content of Arg, Glu, Ila, and Asp is high, while the content of Gys, Leu, Thr, and Gly is low.

**Table 1:** Amino acid composition of Sacha Inchi protein.

Amino acid	Content(%)
Arg	22.98
Glu	11.07
Ile	8.19
Asp	7.95
Tyr	7.81
Pro	7.75
Ser	5.81
Met	5.04
Lys	3.16
Val	3.10
Ala	2.83
His	2.76
Phe	2.80
Cys	2.45
Leu	2.36
Thr	1.98
Gly	1.96

### 3.2 Differential scanning calorimetry

**Figure 1:**
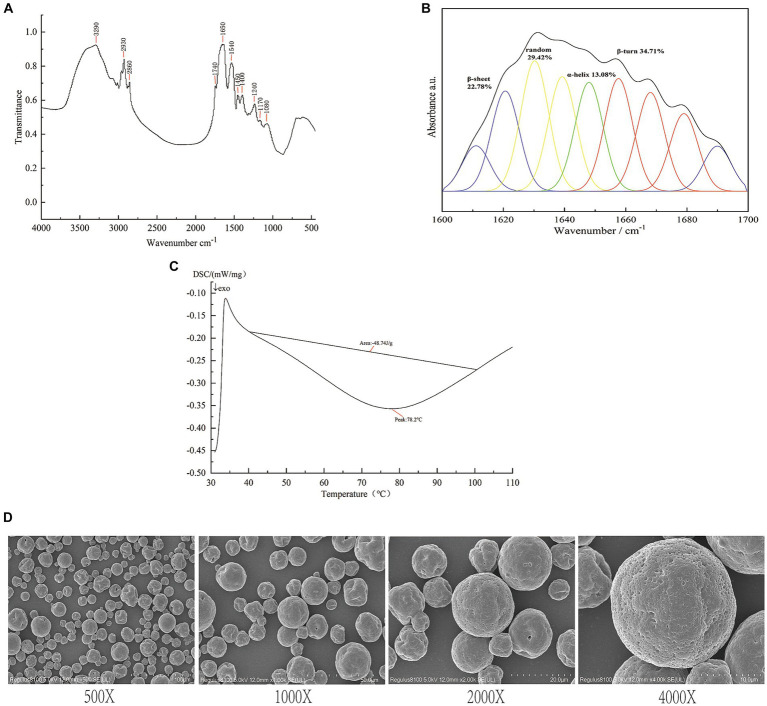
Structure identification of SIP. **(A)** DSC analysis of SIP. **(B)** Infrared spectra of SIP. **(C)** Secondary structure fitting analysis of SIP. **(D)** SEM images of SIP.

The thermal properties of SIP were determined using differential scanning calorimetry (DSC) (32). The denaturation temperature corresponds to the peak temperature of the spectrum, which represents the thermal stability of the protein. The enthalpy value is determined by the peak area of the highest peak, reflecting the degree of protein molecular aggregation. The protein deformation process is endothermic, typically an irreversible system change, with reaction enthalpies ranging from 100 kJ/mol to 400 kJ/mol. DSC is commonly used to detect protein deformation based on the endothermic signals in the range of 40–100°C. Figure 1A shows the DSC curve of the SIP sample, and the results indicate an endothermic peak temperature of 78.2°C and a reaction enthalpy of 48.74 J/g in the 40–100°C range.

### 3.3 FT-IR analysis of SIP

Fourier-transform infrared (FT-IR) spectroscopy is the most commonly used method for protein structure analysis. In the SIP sample, the peak at 3290 cm^−1^ corresponds to the stretching vibration of O-H and N-H bonds, while the peaks at 29330 cm^−1^ and 2,860 cm^−1^ represent the stretching vibration of C-H bonds. The peak at 1740 cm^−1^ corresponds to the stretching vibration of carbonyl groups (C=O) in esters. Furthermore, peaks at 1650 cm^−1^, 1,540 cm^−1^, and 1,240 cm^−1^ correspond to the vibrational modes of protein amide I, II, and III bands, respectively. The peaks at 1450 cm^−1^ and 1,400 cm^−1^ indicate the bending vibration of C-H bonds, while the peaks at 1170 cm^−1^ and 1,080 cm^−1^ mainly correspond to the stretching vibration of C-O-C bonds (Figure 1B).

The protein secondary structure is mainly composed of α-helices, β-sheets, β-turns, and random coils. α-Helices indicate the regular arrangement of protein molecules, while β-sheets and turns reflect the flexibility of protein molecules (33). To investigate the spatial conformation of SIP, we performed a fitting analysis of the protein secondary structure. The results, as shown in Figure 1C, indicate that β-turns are the dominant secondary structure in SIP, accounting for 34.71%. This is followed by random coils and β-sheets, accounting for 29.42 and 22.78%, respectively, suggesting a relatively low degree of molecular aggregation and a more relaxed structure within SIP.

### 3.4 Scanning electron microscope analysis

Scanning electron microscopy (SEM) is a widely used tool for observing the surface morphology of proteins at the molecular level. Under magnifications of 400x, 1,000x, 2000x, and 4,000x, SEM images of SIP reveal distinct morphological features (Figure 1D). SIP appears in a granular form with non-uniform particle sizes. Most protein particles have a smooth surface, while a small portion of particles exhibit surface depressions. This may be attributed to the disruption of hydrogen bonds in some proteins during the spray drying process, leading to changes in protein secondary structure and the inward collapse of protein particles, resulting in surface depressions.

### 3.5 Effect of SIP on mouse fecal metabolome

**Figure 2:**
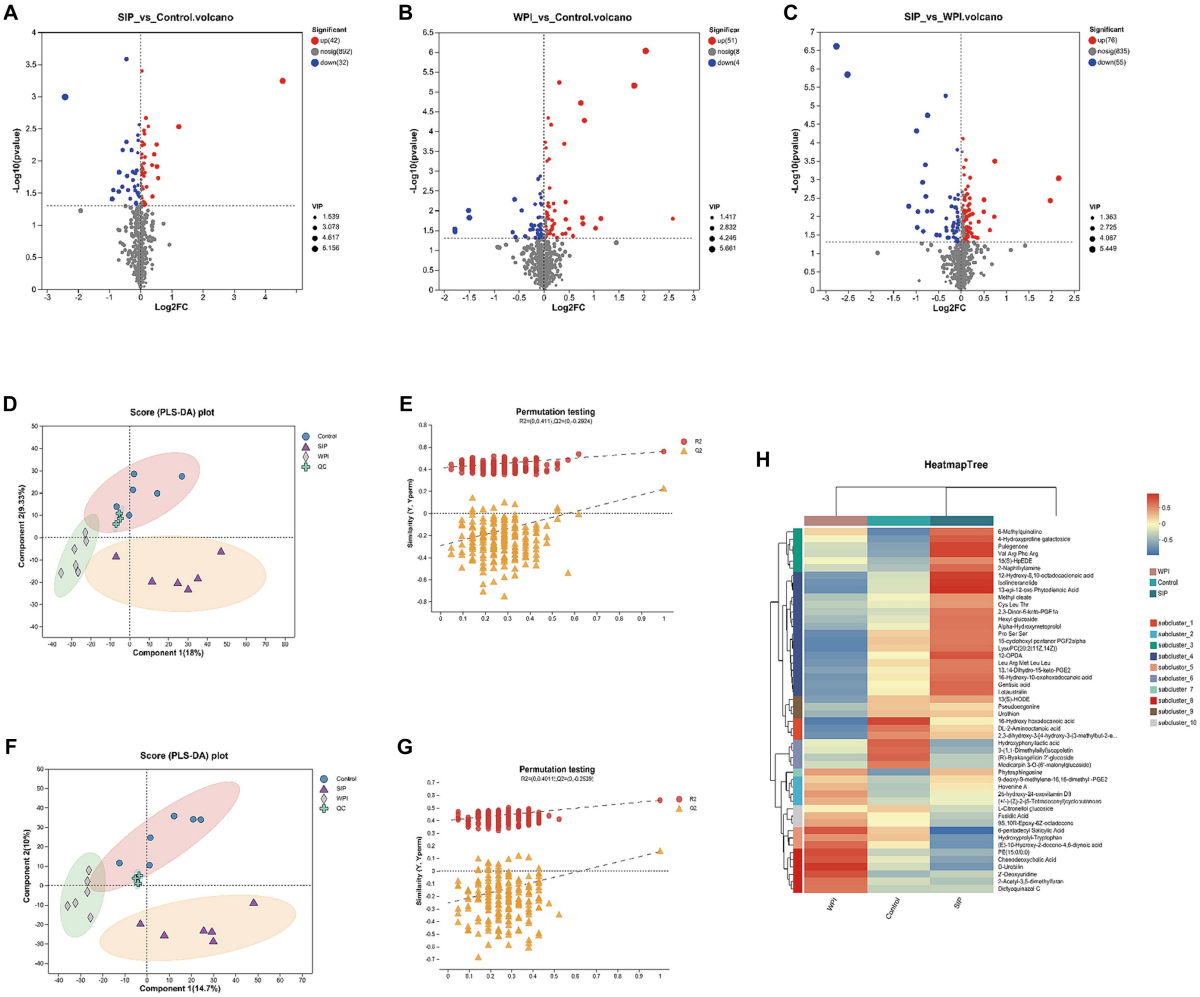
Metabolomic variation after SIP or WPI supplementation. **(A–C)** Volcano plots of differential metabolites (positive and negative ion combined) between different groups, and the red dots were metabolites that *p*-value<0.05 and VIP-pred-OPLS-DA > 1. The blue dots are metabolites with down-regulated expression, and the red dots are metabolites with up-regulated expression, and the points farther to the left and right and the upper edge, the more significant the expression differences. **(D,F)** PLS-DA score plot. **(E,G)** PLS-DA permutation testing. **(D)** is for positive ion combined analysis, **(E)** is for negative ion analysis. **(H)** The heat maps reflect the differences in metabolites between the Control, SIP, and WPI groups (high levels in red, low levels in blue).

The impact of SIP and WPI on the fecal metabolome of C57Bl/6 J mice was assessed using UHPLC–MS/MS. In the cation mode, a total of 815 compounds were detected, and 160 metabolites were identified. In the anion mode, 1,036 compounds were detected, and 61 metabolites were identified. Volcano plot analysis was performed to differentiate significantly upregulated and downregulated metabolites between the control group and the SIP/WPI groups. As shown in Figure 2A, the volcano plot revealed 74 metabolites with significant differences (value of *p* <0.05, VIP-pred >1) between the SIP group and the control group, including 42 upregulated metabolites and 32 downregulated metabolites (Supplementary Table S1). In Figure 2B, the WPI group exhibited significant differences compared to the control group, with 92 metabolites showing upregulation and 41 metabolites showing downregulation (Supplementary Table S2). Figure 2C demonstrated significant differences in metabolites between the SIP and WPI groups, with 131 metabolites being upregulated and 55 metabolites being downregulated (Supplementary Table S3).

Partial least squares discriminant analysis (PLS-DA) is a supervised statistical method used to establish a relationship model between metabolite expression levels and sample categories, enabling the prediction of sample categories and maximizing differentiation between groups. PLS-DA analysis was performed on the selected variables to evaluate their ability to discriminate between the experimental and control groups. A permutation test was conducted to validate the model. The criterion for the evaluation of the permutation test is to look at the intercept between the Q2 regression line and the Y-axis. If the intercept is less than 0.05, the model is robust and reliable, and no overfitting occurs. Distinct separation among the control, SIP, and WPI groups was observed under cation conditions (Figure 2D). The validation results of the PLS-DA model showed a Q2 regression line intercept of −0.2924 (<0.05), indicating a robust and reliable model without overfitting (Figure 2E). Similar results were obtained under anion conditions (Figures 2F, G).

To illustrate the relationship between samples and the differential expression of metabolites among different samples, hierarchical cluster analysis (HCA) was performed on the expression levels of the top 50 significantly different metabolites. The clustering results were visualized in the form of a heat map. As shown in Figure 2H, both SIP and WPI had a significant impact on mouse metabolism. Compared to the control group, the SIP group exhibited significant upregulation of metabolites such as LysoPC (20:2(11Z,14Z)), Val Arg Phe Arg, Pulegenone, 12-oxo-phytodienoic acid (12-OPDA), and Gentisic acid, while metabolites such as Chenodeoxycholic Acid, Hydroxyprolyl-Tryptophan, (E)-10-Hydroxy-2-decene-4,6-diynoic acid, and D-Urobilin were downregulated. In the WPI group, metabolites including D-Urobilin, 2’-Deoxyuridine, Chenodeoxycholic Acid, and 6-pentadecyl Salicylic Acid were upregulated, while metabolites such as 16-Hydroxy hexadecanoic acid, DL-2-Aminooctanoic acid, LysoPC (20:2(11Z,14Z)), and 15-cyclohexyl pentanor PGF2alpha were downregulated.

### 3.6 Changes in KEGG pathways

**Figure 3:**
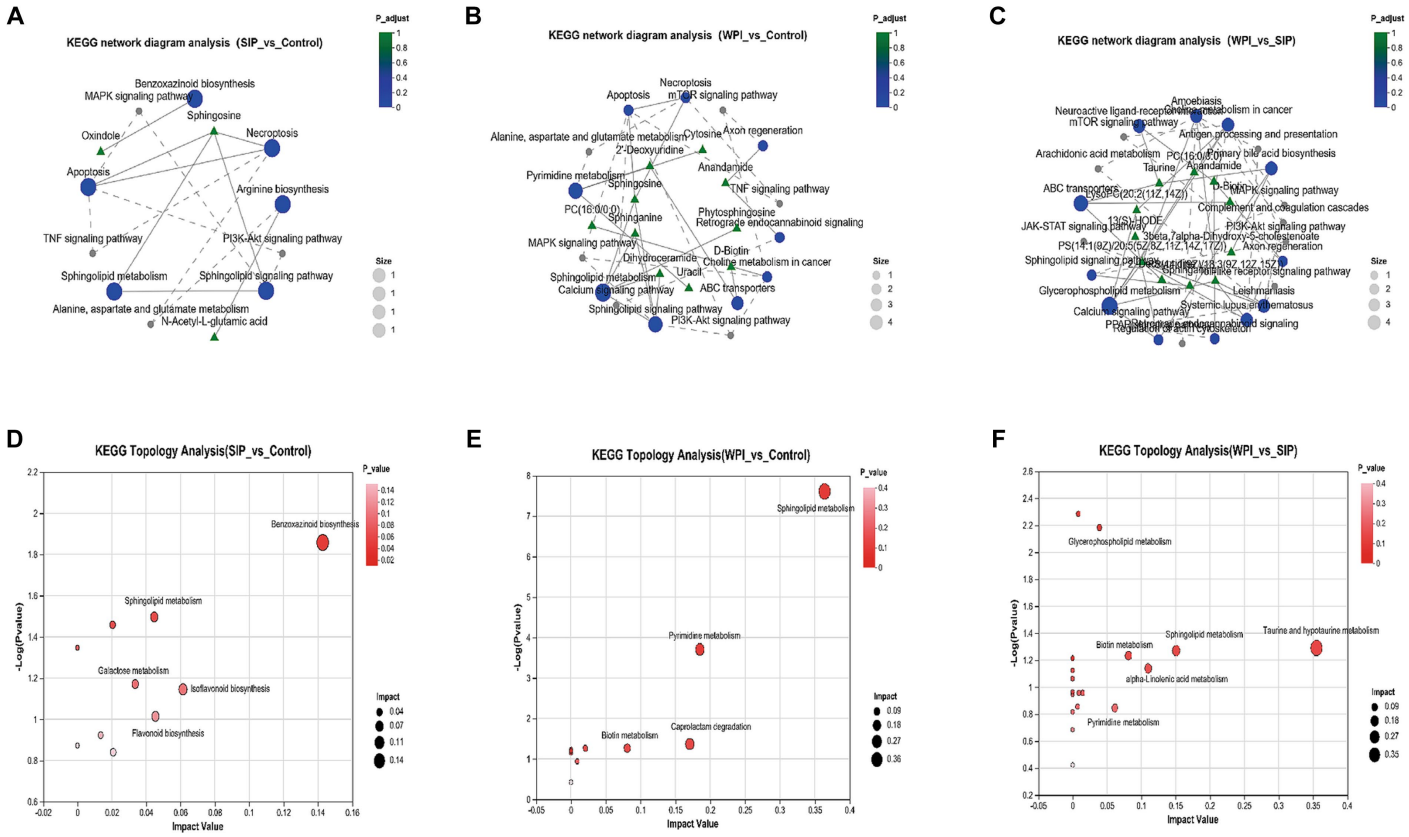
**(A–C)** KEGG network diagram analysis among different groups. The green triangular nodes shown here represent metabolites; The blue circular nodes represent the detected KEGG pathways, and the gray nodes represent the additional pathways from the KEGG connection database. The solid lines in the figure indicate that there is a connection between the paths; The dashed line represents the connection line connecting the supplementary path to the input path, forming a network of relationships. **(D–F)** The bubble map shows the pathway of significant enrichment of differentiated metabolites in feces of different experimental groups. Each bubble in the figure represents a KEGG Pathway; The horizontal axis represents the magnitude of the Impact Value of the relative importance of metabolites in the pathway; The vertical axis represents the enrichment significance of metabolite participation pathway −log10(*p* value); Bubble size represents Impact Value. The larger the bubble, the more important the path.

Metabolomic analysis not only reveals changes in individual metabolites but also provides comprehensive insights into alterations in metabolic pathways induced by exogenous compounds. To further characterize metabolic changes, the significantly altered metabolites were further analyzed using KEGG Pathway Version 1.0.0 to reveal changes in metabolic pathways induced by SIP and WPI. Figures 3A–C represent KEGG network diagrams, which visually display the relationships between KEGG pathways and metabolites. Only pathways with a value of *p* <0.05 were selected. The value of *p* represents the statistical significance of the enrichment results, whereas a smaller value of *p* indicates higher statistical significance. Generally, a value of *p* below 0.05 is considered a significantly enriched function. The results demonstrated that SIP significantly influenced metabolic pathways such as Apoptosis, Necroptosis, Sphingolipid signaling pathway, Sphingolipid metabolism, Benzoxazinoid biosynthesis, and Arginine biosynthesis, compared to the control group. The metabolite Sphingosine played a vital role, involving changes in four metabolic pathways (Figure 3A). WPI exerted significant effects on Sphingolipid metabolism, Sphingolipid signaling pathway, Pyrimidine metabolism, and Apoptosis, among other metabolic pathways. Unlike the SIP group, the WPI group exhibited changes in a broader range of metabolic pathways with corresponding increases in associated metabolites, but Sphingosine remained the most important metabolite (Figure 3B). Additionally, a comparison between WPI and SIP revealed significant changes in metabolic pathways such as the Sphingolipid signaling pathway, Choline metabolism in cancer, ABC transporters, Amoebiasis, and Glycerophospholipid metabolism (Figure 3C). Most of these pathways are associated with human diseases.

The KEGG topological analysis results indicated that, compared to the control group, SIP treatment altered 10 metabolic pathways, including Benzoxazinoid biosynthesis, Isoflavonoid biosynthesis, Sphingolipid metabolism, Flavonoid biosynthesis, Arginine biosynthesis, and Galactose metabolism (Figure 3D). Following WPI treatment, 10 metabolic pathways showed alterations, including Sphingolipid metabolism, Pyrimidine metabolism, Caprolactam degradation, and Biotin metabolism (Figure 3E). Compared to SIP, WPI treatment resulted in changes in 19 metabolic pathways, including Taurine and hypotaurine metabolism, Sphingolipid metabolism, alpha-linolenic acid metabolism, Biotin metabolism, Pyrimidine metabolism, and Glycerophospholipid metabolism (Figure 3F). These metabolic pathways are strongly associated with lipid metabolism, xenobiotic biodegradation and metabolism, amino acid metabolism, and the biosynthesis of secondary metabolites.

### 3.7 SIP and WPI adjusted the gut microbiome distribution of intestinal flora in mice

**Figure 4:**
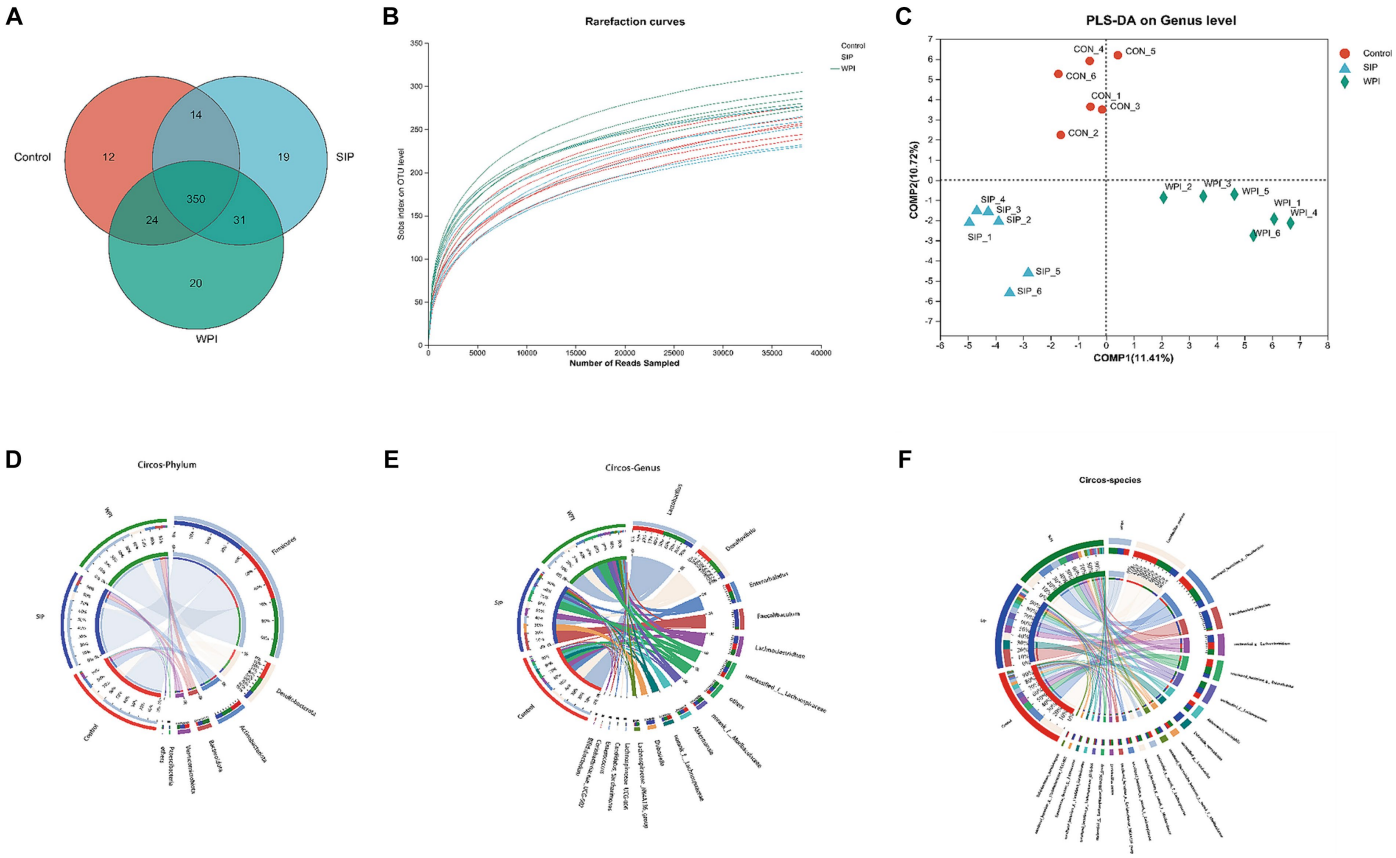
SIP and WPI changed the composition of intestinal flora in mice. **(A)** Venn diagram of OTUs in the three treatments. **(B)** Rarefaction Curve of the three treatments. **(C)** PLS-DA analysis based on genus level. **(D)** Microbial community distribution at phylum level in three groups. **(E)** Microbial community distribution at genus level in three groups. **(F)** Microbial community distribution at species level in three groups. The data was visually processed by Circos. The left half circle represents the composition of species in the sample, the color of the outer ribbon represents the group from which the color of the inner ribbon represents the species, and the length represents the relative abundance of the species in the corresponding sample. The right half circle represents the distribution proportion of species in different samples at the taxonomic level, the outer ribbon represents species, the inner ribbon color represents different groups, and the length represents the distribution proportion of the sample in a certain species.

Differences in microbial community structure among the control group, SIP group, and WPI group were characterized by analyzing the 16S rRNA gene sequences from microbial samples isolated from the cecal contents of mice. The overall structural changes in the gut microbiota following SIP and WPI treatments were determined. Sequencing results revealed a total of 1,079,097 raw reads, which were optimized to 988,221 sequences after removing chimeras and low-quality reads. Clustering and species annotation of the clean reads from all samples resulted in 470 valid operational taxonomic units (OTUs). As shown in Figure 4A, all three experimental groups shared 350 OTUs, which can be considered as core microbiota. Additionally, the control, SIP, and WPI groups exhibited 12, 19, and 20 OTUs specific to each respective group.

The dilution curve was constructed by randomly selecting a certain number of sequences from each sample and calculating the corresponding alpha diversity index. The plateauing of the curve indicated sufficient sequencing depth in this study, as the sobs index values reached stability with increasing sample size (Figure 4B). Inter-group similarity analysis was conducted on the grouped samples to test the significance of inter-group differences. At the genus level, Partial Least Squares Discriminant Analysis (PLS-DA) was performed on the gut microbiota communities of each experimental group. The results showed distinct clustering of samples from the three groups, indicating significant differences in gut microbiota composition among them. In other words, both SIP and WPI interventions led to significant changes in the gut microbiota structure of the mice.

Circos plots of the sample-species relationship are a visual representation of the correspondence between samples and species. These plots not only reflect the proportional composition of dominant species in each group but also indicate the distribution of these dominant species across different groups. The results revealed that all sequences were classified into 10 phyla and 138 genera. As shown in Figure 4D, different groups exhibited similar community structures at the phylum level. The most dominant phyla in the gut microbiota were *Firmicutes*, *Desulfobacterota, Actinobacteriota, Bacteroidetes,* and *Verrucomicrobiota*. *Firmicutes* had the highest relative abundance in all three groups, accounting for 61% in the control group, 72% in the SIP group, and 60% in the WPI group. This indicates a significant increase in *Firmicutes* abundance following SIP treatment. Compared to the control group, the SIP group showed a significant reduction in the abundance of *Desulfobacterota* (18% in control, 8.8% in SIP, 17% in WPI) and *Actinobacteriota* (12% in control, 7.6% in SIP, 11% in WPI), while no significant changes were observed in the WPI group. Additionally, both SIP and WPI treatments significantly increased the abundance of *Bacteroidetes* (1.8% in control, 5.8% in SIP, 7.6% in WPI).

At the genus level, the 16 most abundant genera were *Lactobacillus*, *Desulfovibrio*, *Enterorhabdus*, *Faecalibaculum*, *Lachnoclostridium*, *unclassified_f_Lachnospiraceae, norank_f_Muribaculaceae*, *Akkermansia*, *norank_f_Lachnospiraceae*, *Dubosiella*, *Lachnospiraceae_NK4A136_group*, *Lachnospiraceae_UCG-006*, *Candidatus_Saccharimonas*, *Enterococcus*, *Coriobacteriaceae_UCG-002*, *Bifidobacterium*. Microbial community structure analysis was performed on the top 16 genera in each group (Figure 4E). The results showed that the dominant genera in the control group were *Lactobacillus* (34%), *Desulfovibrio* (18%), *Enterorhabdus* (10%), and *Lachnoclostridium* (8.3%). In the SIP group, the dominant genera were *Faecalibaculum* (22%), *Dubosiella* (11%), *Lactobacillus* (9.8%), and *Lachnoclostridium* (9.2%). The dominant genera in the WPI group were *Lactobacillus* (26%), *Desulfovibrio* (17%), *Enterorhabdus* (10%), and *g-unclassified-f-Lachnospiraceae* (7.9%). The dominant genera in the control and WPI groups were similar, while there were significant changes in the dominant genera in the SIP group. Compared to the control group, SIP treatment significantly reduced the levels of *Lactobacillus*, *Desulfovibrio*, and *Enterorhabdus*, while increasing the levels of *Faecalibaculum* and *Dubosiella*. WPI treatment decreased the levels of *Faecalibaculum* and *Akkermansia* while increasing the levels of *Faecalibaculum* and *g-norank-f-Muribaculaceae*. It is worth mentioning that *Dubosiella* was detected at very low levels in the control group and was not detected in the WPI group, indicating that it may be a unique genus in the SIP-treated group.

The microbial community structure was analyzed at the species level for the 21 most abundant species (Figure 4F). The results showed significant changes in the intestinal flora of mice under different treatment groups. The abundance of *Lactobacillus_murinus* and *Bifidobacterium_pseudolongum* was reduced in the SIP and WPI groups compared to the control group (*p* < 0.05). *Faecalibaculum_rodentium* was detected in extremely high abundance in the SIP *group* (*p* < 0.001) and slightly increased in the WPI group (*p* < 0.05).*Dubosiella_newyorkensis* was detected only in the SIP group. *Lactobacillus_reuteri* was decreased in SIP (*p* < 0.05) and increased in WPI (*p* < 0.05).

### 3.8 Correlation analysis between differential metabolites and intestinal flora

Correlation analysis is used to analyze the relationship between two or more variables that show correlation, thereby measuring the degree of correlation between the variables. In this study, a correlation analysis of metabolites and gut microbiota was conducted based on the Pearson correlation coefficient. The microbiota was selected at the phylum level, and the metabolomic data underwent dimensionality reduction (see Supplementary Table). Dimensionality reduction helps eliminate redundant features and facilitates the discovery of metabolite/microbiota features with similar expression profiles. Hierarchical clustering (HCLUST) was used to analyze the correlation between differential metabolites and gut microbiota.

**Figure 5:**
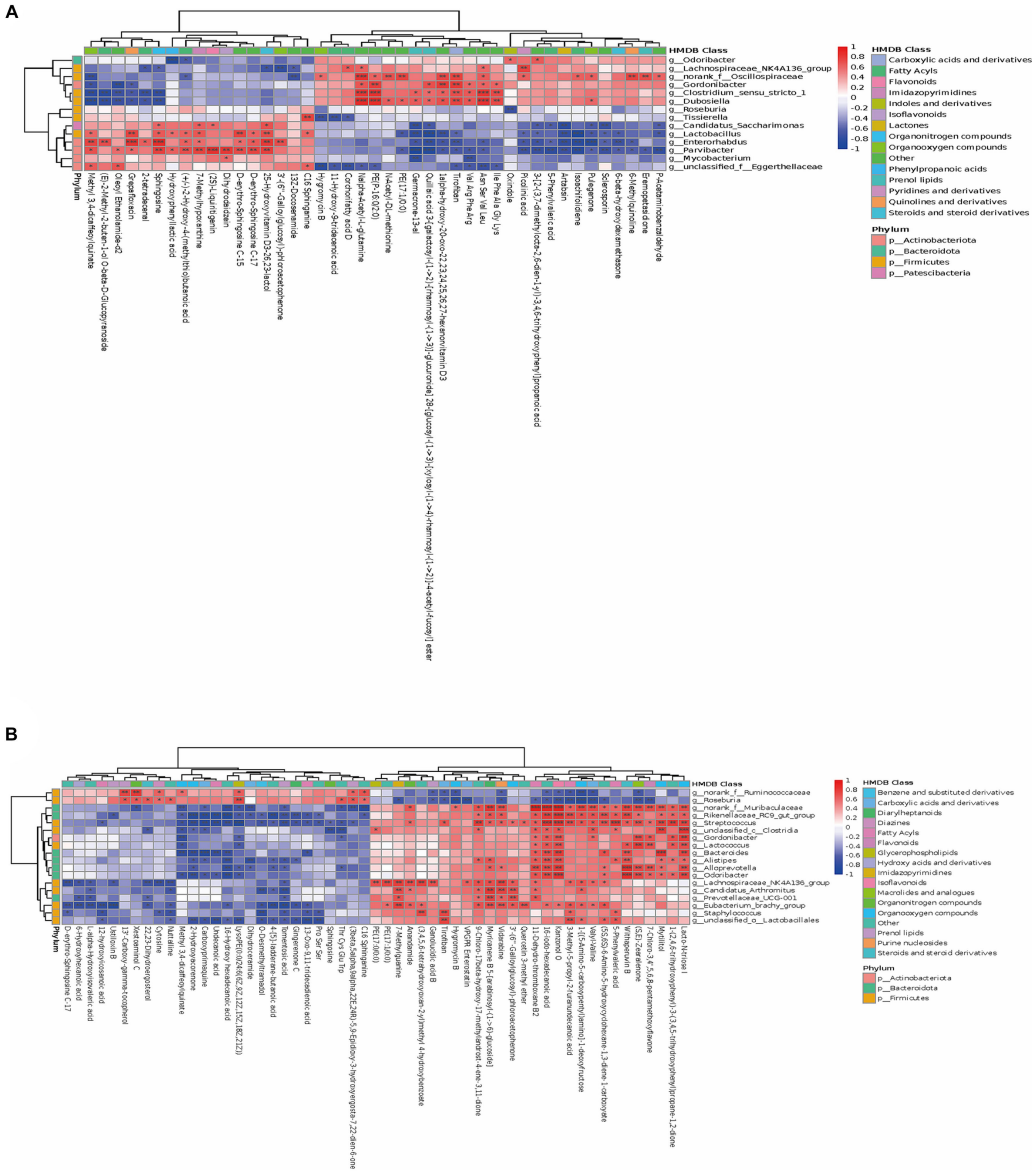
The close correlations between the relative abundance of gut microbiota and differential metabolites. **(A,B)** The close correlations between the relative abundance of the main genus in the gut microbiota and the differential metabolites between the SIP and Control group, WPI and Control group. On the right side of the heat map are the microbial categories (phylum level) and on the lower side are metabolites. The left and the top of the cluster tree are made according to the correlation coefficient. The red represents positive correlation, while the blue represents negative correlation, and the darker the color, the stronger the correlation. In addition, HMDB compound classification and phylum level classification were, respectively, made for the differential metabolites and the differential flora, which were represented by different color blocks in the legend (* *p*-value < 0.05, ** *p*-value < 0.01, *** *p*-value < 0.001).

The results showed a strong correlation between certain bacteria and various metabolites at the phylum level. These bacteria included *Firmicutes*, *Actinobacteriota*, *Patescibacteria*, and *Bacteroidota*. In terms of potential biomarkers, Tirofiban, Sphingosine, Ile Phe Ala Gly Lys, 16-iodo-hexadecanoic acid, 11-Dehydro-thromboxane B2, Kanzonol O, and others were closely related to gut microbiota (Figure 4). As shown in Figure 5A, at the genus level, *Dubosiella*, *Gordonibacter*, and *Clostridium-sensu-stricto-1* showed significant positive correlations with Tirofiban, Ile Phe Ala Gly Lys, while exhibiting significant negative correlations with Grepafloxacin and Oleoyl Ethanolamide-d2. *Parvibacter*, *Enterorhabdus*, and *Lactobacillus* exhibited significant positive correlations with Sphingosine and Grepafloxacin while showing significant negative correlations with Artabsin, Isoachifolidiene, and Pulegenone. As shown in Figure 5B, *Muribaculaceae*, *Streptococcus*, exhibited significant positive correlations with Kanzonol O, Lacto-N-triose I, while showing significant negative correlations with 16-Hydroxy hexadecanoic acid and Tomentosic acid.

### 3.9 KEGG path analysis

We annotated KEGG pathways for differential metabolites, predicted KEGG function in the microbiome, obtained commonly involved pathways, counted and clustered the abundance of the pathways, and obtained a visual heat map, as shown in Supplementary Figure S1. As shown in Supplementary Figure S1, in terms of bacterial flora, Limonene and pinene degradation, Insect hormone biosynthesis, Arachidonic acid metabolism, Calcium signaling pathway, and Apelin signaling pathway were increased and the Degradation of aromatic compounds, Glycerophospholipid metabolism, Tyrosine metabolism, and Amoebiasis were increased in SIP group as compared to the control. The apelin signaling pathway increased in abundance and Degradation of aromatic compounds, Glycerophospholipid metabolism, Tyrosine metabolism, and Amoebiasis decreased in abundance. In terms of metabolites, Ubiquinone and other terpenoid-quinone biosynthesis, Pantothenate and CoA biosynthesis, Flavone and flavonol biosynthesis, and other pathways were reduced in the SIP group. Biosynthesis, and Apoptosis, Necroptosis, and Galactose metabolism pathways increased in abundance, and Apoptosis, Necroptosis, and Galactose metabolism pathways decreased in abundance.

## 4 Discussion

Protein is essential for the body’s vital activities as it not only participates in tissue building but also regulates various metabolic pathways and immune system activity. In this study, we extracted SIP from the Sacha Inchi seed to investigate its amino acid composition and basic structure. We examined its effects on the fecal metabolomics and cecal microbiota diversity of healthy C57/BL6 mice and compared it with animal-derived protein whey protein isolate (WPI), exploring the possibility of replacing or partially substituting animal protein with plant-based protein SIP.

The functional characteristics of proteins depend on their structural features, including amino acid composition and sequence, molecular size and configuration, as well as physicochemical properties (3). According to our results, SIP is rich in amino acid content and diverse in types. Among them, arginine is the most abundant amino acid, accounting for 22.98% of the total amino acid content. As a non-essential amino acid in nutrition, arginine also plays important physiological roles. The role of arginine in cardiovascular disease and metabolic disorders has been extensively studied. One study showed that arginine can affect the differentiation of fat cells, with a beneficial mechanism involving the balance of energy intake and expenditure, favoring the reduction of fat or the decrease in the growth of white adipose tissue, thus treating obesity or its consequences (34). Moreover, studies have reported the protective role of asymmetric dimethylarginine (ADMA) in atherosclerotic vascular disease and the potential use of arginine and other potential nutrients in targeted therapy for cancer and other diseases (35–37). Isoleucine is the most abundant essential amino acid in SIP, and Ile, as a branched-chain amino acid, plays a crucial role in various physiological functions such as growth, immunity, protein metabolism, fatty acid metabolism, and glucose transport (38).

EAAs, as important nutritional signals, regulate human energy balance through various mechanisms, and supplementing certain EAAs is recognized as an intervention measure for weight reduction (39). It has been reported that the intake of methionine (Met), valine (Val), and leucine (Leu) can reduce body weight under certain conditions. Additionally, threonine (Thr) has been reported to reduce fat accumulation in obese mice by increasing the expression of lipolysis-related proteins to regulate lipid metabolism (40–43). Furthermore, our study showed that SIP contains lysine (Lys) and Thr, which are generally considered to be lacking in plant proteins. It may be due to the lack of Trp in SIP due to some reasons during the extraction process, and the total EAAs content is lower than the amino acid requirements in the FAO/WHO/UNU Expert Consultation (44). Therefore, when used as the sole source of protein, the requirements for essential amino acids cannot be met, and it needs to be used in combination with other proteins (45).

In this study, we analyzed the impact of SIP on the fecal metabolome of mice through LC/MS analysis. From the results, SIP mainly affected lipid metabolism, energy expenditure, and absorption, as well as the synthesis and metabolism of amino acids such as arginine (Arg), methionine (Met), and cysteine (Cys). Arg has been proven to have beneficial effects in reducing obesity (34), and Met can reduce endogenous fat production, increase lipid breakdown metabolism, and have anti-diabetic and lipid-lowering effects (44). Cys has been found to stimulate cell protection by reducing the production of reactive oxygen species and ameliorating apoptosis induced by doxorubicin and is considered to have the potential for antioxidant stress and tissue damage (46). LysoPC(20:2(11Z,14Z)) is a lysophospholipid (LyP) that acts in lipid signal transduction by interacting with lysophospholipid receptors (LPL-R), and its potential pro-inflammatory and anti-inflammatory activities have also been reported in the vascular system (47). 12-oxo-phytodienoic acid (12-OPDA) is a plant-derived anti-inflammatory compound. It has been reported that 12-OPDA inhibits inflammation in murine microglia by suppressing Nf-κB and p38 MAPK signaling activated by lipopolysaccharide (LPS) (48). Additionally, Gentisic acid has been reported in biochemical studies for its anti-inflammatory, antimicrobial, antioxidant, and neuroprotective effects (49–53). Sphingosine is a diverse class of lipids, and the sphingolipid family plays an important role in membrane biology and provides biologically active metabolites that regulate cellular functions. It has various functions in mammalian development and physiology. For example, it can alleviate non-alcoholic fatty liver disease (NAFLD) in mice by regulating Sphingosine 1-phosphate receptor signaling (54, 55). In summary, changes including but not limited to the above metabolites were found in our study, suggesting that SIP can regulate a variety of physiological functions by affecting the metabolism of compounds, but the regulatory mechanisms need to be further investigated.

The impact of protein intake on the composition of the intestinal microbiota, and its subsequent regulation of the intestinal flora, has been extensively studied (56, 57). Obese individuals typically display a higher abundance of *Firmicutes* in their intestinal flora, which is considered a contributing factor to obesity, whereas *Bacteroidetes* have been shown to reduce fat accumulation (20, 58). In recent studies, extracts of Coleus forskohlii and *Garcinia indica* were found to mitigate lipid accumulation in obese mice by decreasing the abundance of *Firmicutes* and increasing the abundance of *Bacteroidetes* (59). Therefore, the *Firmicutes/Bacteroidetes* ratio can be regarded as an indicator of obesity to some extent. In our study, SIP decreased the *F/B* value although it increased the abundance of *Firmicutes*, a result that suggests that SIP may have an anti-obesity effect by decreasing the *F/B* value. Notably, the WPI group exhibited an even greater decrease in the *F/B* value compared to SIP. *Actinobacteriota* is an important cellulolytic bacterium involved in the digestion of plant matter and energy provision to the host (60). Studies have reported a higher abundance of *Actinobacteriota* and *Desulfobacterota* in patients with intestinal and psychiatric disorders, and inhibition of *Desulfobacterota* has shown protective effects on dopaminergic (DA) neurons (61). Our results showed that SIP significantly reduced the abundance of *Actinomycetes* and *Desulfovibrio* flora compared to the control and WPI groups, suggesting that SIP may have potentially beneficial effects on intestinal and psychiatric disorders.

*Desulfovibrio* is a gram-negative sulfate-reducing bacterium that is a resident symbiont in the human gastrointestinal tract and has potential roles in various human diseases, including bacteremia, inflammatory bowel disease, neurodegenerative diseases, autism, and metabolic syndrome (62). *Faecalibaculum* can inhibit the growth of intestinal tumor cells by releasing short-chain fatty acids without affecting adapt immune cells (63). *Dubosiella* is a genus of gram-positive bacteria that has been reported to have important protective effects in sepsis-related brain injury. It is also reported to have potential effects in regulating SCFA production and obesity (64). In our study, SIP decreased the abundance of *Desulfovibrio*, a harmful intestinal bacterium, and increased the abundance of *Faecalibaculum*, a beneficial intestinal bacterium, *Dubosiella*. *Faecalibaculum_rodentium* controls eosinophil-dependent intestinal epithelial homeostasis by decreasing retinoic acid signaling required to maintain certain intestinal eosinophil populations by decreasing expression of the retinoic acid-producing enzymes Adh1, Aldh1a1, and Rdh7 in enterocytes (65). *Dubosiella_newyorkensis* has positive effects in reducing oxidative stress, improving vascular endothelial function, and redistributing intestinal flora (65), and its potential anti-aging effects in mice have been demonstrated (66). Therefore, we speculate that SIP may have a potential positive role in gut health and obesity suppression.

Research on Sacha Inchi has focused on Sacha Inchi oil, which has been reported to alleviate intestinal flora dysbiosis and improve hepatic lipid metabolism disorders in rats fed a high-fat diet (67). And in terms of protein, Kun Wang et al. treated Sacha Inchi meal with trypsin to obtain its hydrolyzed products and found that the hydrolyzed products exhibited anti-hyperuricemic activity by reducing renal injury and modulating the intestinal microbiota (68). In addition, there is also information about the protein consumption of sacha inchi flour has the same nitrogen balance as soybean flour. There is also information about protein consumption of sacha inchi flour has the same nitrogen balance as soybean flour (64). In the present study, we found that SIP altered the composition of the intestinal flora in mice and regulated a variety of metabolic activities in the host by affecting the metabolism of small molecule compounds, but the exact mechanism of regulation needs to be further investigated. We have gained a deeper understanding of the role of SIP in the regulation of the mouse metabolome and gut flora by analyzing the pathways in which differential metabolites and gut microorganisms are jointly involved, and we can follow up with more in-depth analyses of the top-ranked pathways and validate the potential health benefits that may be associated with SIP, which is the next step in the work we need to carry out.

## 5 Conclusion

Our research reported significant changes in fecal metabolomic profiles and gut microbiota in mice after ingestion of SIP. Successful identification of multiple metabolic pathways, including lipid metabolism and amino acid metabolism, was achieved through fecal metabolomics. The enrichment analysis of KEGG pathways indicated their relevance to human diseases. In summary, SIP is beneficial to health, and the identified metabolic pathways may serve as key clues for future investigations into the mechanisms underlying SIP’s health-promoting effects.

## Data availability statement

The original contributions presented in the study are included in the article/Supplementary material, further inquiries can be directed to the corresponding author.

## Ethics statement

The animal study was approved by Animal Care Committee of the College of Animal Science and Technology, Yunnan Agricultural University (Ethical Review No. 202209012). The study was conducted in accordance with the local legislation and institutional requirements.

## Author contributions

KW: Investigation, Software, Writing – original draft, Writing – review & editing. WG: Conceptualization, Data curation, Writing – original draft, Writing – review & editing. SL: Data curation, Formal analysis, Writing – review & editing. SH: Investigation, Methodology, Writing – original draft. HM: Formal analysis, Visualization, Writing – original draft. MW: Investigation, Software, Writing – original draft. JS: Funding acquisition, Methodology, Project administration, Writing – review & editing. CZ: Supervision, Writing – review & editing.

## Footnotes

^1^ http://circos.ca/

## Supplementary material

SUPPLEMENTARY FIGURE S1 Heatmap of KEGG pathway visualization with differential metabolite and microbial co-involvement.” It is already cited in the article.
