# Supplementary figures and images for: Regulation of Sacha Inchi protein on fecal metabolism and intestinal microorganisms in mice
Source: Front Nutr. 2024 Mar 8;11:1354486. doi: 10.3389/fnut.2024.1354486 (PMC10959099; doi:10.3389/fnut.2024.1354486)

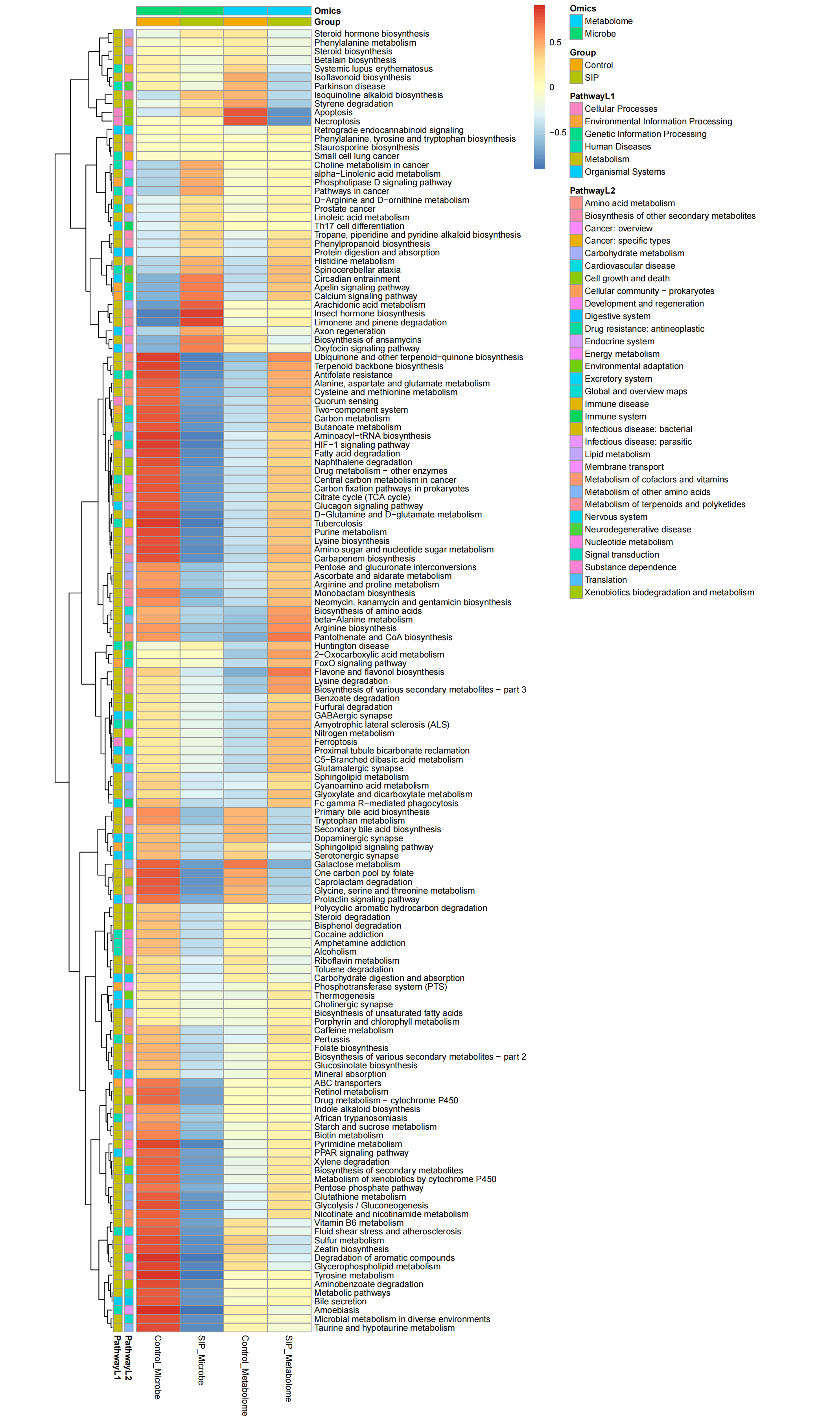

Supplement: SUPPLEMENTARY FIGURE S1 — Heatmap of KEGG pathway visualization with differential metabolite and microbial co-involvement.” It is already cited in the article. [file Image_1.tif]
