# Supplementary material for: Regulation of Sacha Inchi protein on fecal metabolism and intestinal microorganisms in mice
Source: Front Nutr. 2024 Mar 8;11:1354486. doi: 10.3389/fnut.2024.1354486 (PMC10959099; doi:10.3389/fnut.2024.1354486)
Supplement: Supplementary file 2 [file Table_1.DOCX]

Table S1 Significant metabolites differences between the SIP and control groups.

| Metab ID | Metabolite | Log_2_FC | Regulate |
| --- | --- | --- | --- |
| metab_4120 | Tirofiban | 4.5612 | up |
| metab_1357 | Quillaic acid 3-[galactosyl-(1->2)-[rhamnosyl-(1->3)]-glucuronide] 28-[glucosyl-(1->3)-[xylosyl-(1->4)-rhamnosyl-(1->2)]-4-acetyl-fucosyl] ester | 1.2280 | up |
| metab_9860 | Quercetin 3-methyl ether | 0.5687 | up |
| metab_3698 | P-Acetaminobenzaldehyde | 0.5277 | up |
| metab_10305 | Nalpha-Acetyl-L-glutamine | 0.5175 | up |
| metab_3631 | Hygromycin B | 0.4412 | up |
| metab_797 | 2,6,10,10-Tetramethyl-1-oxaspiro[4.5]decan-6-ol | 0.3828 | up |
| metab_5022 | (4-ethyl-2,6-dihydroxyphenyl)oxidanesulfonic acid | 0.3725 | up |
| metab_7878 | 5-Phenylvaleric acid | 0.2506 | up |
| metab_8662 | Lycopersiconolide | 0.1975 | up |
| metab_484 | 6-Methylquinoline | 0.1754 | up |
| metab_1572 | Oxindole | 0.1589 | up |
| metab_3195 | Sclerosporin | 0.1524 | up |
| metab_3054 | Germacrone-13-al | 0.1447 | up |
| metab_8134 | Sclareol | 0.1391 | up |
| metab_6727 | PE(17:1/0:0) | 0.1325 | up |
| metab_1571 | 6-[2-(2H-1,3-benzodioxol-5-yl)ethyl]-4-hydroxy-5,6-dihydro-2H-pyran-2-one | 0.1274 | up |
| metab_8434 | 15(S)-HpEDE | 0.1188 | up |
| metab_3227 | 6-beta-hydroxydexamethasone | 0.1166 | up |
| metab_1686 | Isoachifolidiene | 0.1120 | up |
| metab_5215 | N-Acetyl-DL-methionine | 0.1094 | up |
| metab_10285 | N-Acetyl-L-glutamic acid | 0.1082 | up |
| metab_3168 | Isolinderanolide | 0.1081 | up |
| metab_2783 | PE(P-16:0/2:0) | 0.0932 | up |
| metab_3742 | Pulegenone | 0.0918 | up |
| metab_6439 | (3beta,17alpha,23S)-17,23-Epoxy-3,29-dihydroxy-27-norlanost-8-en-24-one | 0.0830 | up |
| metab_1472 | Artabsin | 0.0801 | up |
| metab_4939 | Galactosyl 4-hydroxyproline | 0.0792 | up |
| metab_4168 | N-Acetylmannosamine | 0.0595 | up |
| metab_1570 | 1alpha-hydroxy-20-oxo-22,23,24,25,26,27-hexanorvitamin D3 | 0.0592 | up |
| metab_364 | THTC | 0.0585 | up |
| metab_920 | Val Arg Phe Arg | 0.0584 | up |
| metab_585 | Eremopetasidione | 0.0533 | up |
| metab_406 | 4-Hydroxyproline galactoside | 0.0520 | up |
| metab_579 | Pro Ala His Arg | 0.0514 | up |
| metab_3390 | 3-[2-(3,7-dimethylocta-2,6-dien-1-yl)-3,4,6-trihydroxyphenyl]propanoic acid | 0.0509 | up |
| metab_3534 | 11-Hydroxy-9-tridecenoic acid | 0.0468 | up |
| metab_722 | Arg Arg Arg Pro | 0.0456 | up |
| metab_4158 | Picolinic acid | 0.0410 | up |
| metab_3334 | Asn Ser Val Leu | 0.0391 | up |
| metab_707 | Corchorifatty acid D | 0.0331 | up |
| metab_942 | Ile Phe Ala Gly Lys | 0.0325 | up |
| metab_3256 | C16 Sphinganine | -0.0386 | down |
| metab_1946 | Sphingosine | -0.0402 | down |
| metab_3868 | Cyclic N-Acetylserotonin glucuronide | -0.0728 | down |
| metab_1086 | Linoleoyl Ethanolamide | -0.0751 | down |
| metab_1124 | Oleoyl Ethanolamide | -0.0758 | down |
| metab_8444 | 25-Hydroxyvitamin D3-26,23-lactol | -0.0760 | down |
| metab_4116 | 7-Methylhypoxanthine | -0.0838 | down |
| metab_3067 | D-erythro-Sphingosine C-17 | -0.0850 | down |
| metab_4394 | Serylhistidine | -0.0867 | down |
| metab_8471 | Albigenic acid | -0.1055 | down |
| metab_1195 | 13Z-Docosenamide | -0.1219 | down |
| metab_1522 | 2,3,4,5,6,7-Hexahydro-6,7-dimethylcyclopent[b]azepin-8(1H)-one | -0.1228 | down |
| metab_1043 | 2-tetradecenal | -0.1442 | down |
| metab_3430 | Alectrol | -0.1612 | down |
| metab_1159 | Oleoyl Ethanolamide-d2 | -0.1684 | down |
| metab_1830 | D-erythro-Sphingosine C-15 | -0.1709 | down |
| metab_9668 | (E)-2-Methyl-2-buten-1-ol O-beta-D-Glucopyranoside | -0.1895 | down |
| metab_5817 | Dihydrodaidzein | -0.2384 | down |
| metab_1812 | 16-Hydroxy hexadecanoic acid | -0.2437 | down |
| metab_3551 | (2S)-Liquiritigenin | -0.2874 | down |
| metab_10067 | 5-hydroxy-1H-indole-3-carboxylic acid | -0.3283 | down |
| metab_9959 | Hydroxyphenyllactic acid | -0.3992 | down |
| metab_4658 | 2-Keto-3-deoxy-D-gluconic acid | -0.4288 | down |
| metab_9655 | Medicarpin 3-O-(6'-malonylglucoside) | -0.4495 | down |
| metab_5201 | (+/-)-2-Hydroxy-4-(methylthio)butanoic acid | -0.4497 | down |
| metab_4843 | 3'-(6''-Galloylglucosyl)-phloroacetophenone | -0.5703 | down |
| metab_7033 | 6-pentadecyl Salicylic Acid | -0.5823 | down |
| metab_1008 | 2-tridecenal | -0.6859 | down |
| metab_488 | Grepafloxacin | -0.6969 | down |
| metab_5624 | Indolelactic acid | -0.8730 | down |
| metab_1526 | (R)-Byakangelicin 2'-glucoside | -0.9083 | down |
| metab_4159 | Methyl 3,4-dicaffeoylquinate | -2.4189 | down |
