# Supplementary material for: Regulation of Sacha Inchi protein on fecal metabolism and intestinal microorganisms in mice
Source: Front Nutr. 2024 Mar 8;11:1354486. doi: 10.3389/fnut.2024.1354486 (PMC10959099; doi:10.3389/fnut.2024.1354486)
Supplement: Supplementary file 3 [file Table_2.DOCX]

Table S2 Significant metabolites differences between the WPI and control groups.

| Metab ID | Metabolite | Log_2_FC | Regulate |
| --- | --- | --- | --- |
| metab_4120 | Tirofiban | 2.5852 | up |
| metab_1339 | 16-iodo-hexadecanoic acid | 2.0414 | up |
| metab_4309 | 1-[(5-Amino-5-carboxypentyl)amino]-1-deoxyfructose | 1.8116 | up |
| metab_2164 | Ganolucidic acid B | 1.1433 | up |
| metab_9829 | Suberylglycine | 1.0365 | up |
| metab_3859 | Valyl-Valine | 0.8123 | up |
| metab_3820 | 1-(2,4,6-trihydroxyphenyl)-3-(3,4,5-trihydroxyphenyl)propane-1,2-dione | 0.7821 | up |
| metab_3990 | Withaperuvin B | 0.7723 | up |
| metab_4108 | Kanzonol O | 0.7404 | up |
| metab_9278 | 3-Hydroxyphenyl-valeric acid | 0.5834 | up |
| metab_9860 | Quercetin 3-methyl ether | 0.5054 | up |
| metab_3970 | Cyclolinopeptide F | 0.4403 | up |
| metab_3312 | Phytosphingosine | 0.4370 | up |
| metab_3631 | Hygromycin B | 0.4348 | up |
| metab_7878 | 5-Phenylvaleric acid | 0.4091 | up |
| metab_1650 | Dictyoquinazol C | 0.3734 | up |
| metab_1322 | 9-Chloro-17beta-hydroxy-17-methylandrost-4-ene-3,11-dione | 0.3078 | up |
| metab_4111 | Lacto-N-triose I | 0.2627 | up |
| metab_4843 | 3'-(6''-Galloylglucosyl)-phloroacetophenone | 0.2171 | up |
| metab_1393 | (S,E)-Zearalenone | 0.2100 | up |
| metab_3234 | Dihomo-gamma-Linolenoyl ethanolamide | 0.1927 | up |
| metab_3019 | (Z)-7-Hexadecen-1,16-olide | 0.1899 | up |
| metab_6727 | PE(17:1/0:0) | 0.1693 | up |
| metab_4033 | Tragopogonsaponin G | 0.1679 | up |
| metab_4146 | Hydroxyprolyl-Proline | 0.1643 | up |
| metab_6509 | PE(13:0/0:0) | 0.1533 | up |
| metab_4155 | 3-Methyl-5-propyl-2-furanundecanoic acid | 0.1437 | up |
| metab_4888 | 7-Chloro-3,4',5,6,8-pentamethoxyflavone | 0.1305 | up |
| metab_10285 | N-Acetyl-L-glutamic acid | 0.1242 | up |
| metab_4152 | (5S,6S)-6-Amino-5-hydroxycyclohexane-1,3-diene-1-carboxyate | 0.1153 | up |
| metab_1571 | 6-[2-(2H-1,3-benzodioxol-5-yl)ethyl]-4-hydroxy-5,6-dihydro-2H-pyran-2-one | 0.1097 | up |
| metab_6661 | PE(16:1/0:0) | 0.1077 | up |
| metab_4138 | Mytilitol | 0.0980 | up |
| metab_4954 | 2'-Deoxyuridine | 0.0894 | up |
| metab_4435 | 7-Methylguanine | 0.0868 | up |
| metab_4154 | Vidarabine | 0.0858 | up |
| metab_3538 | VPGPR Enterostatin | 0.0852 | up |
| metab_1332 | (3,4,5,6-tetrahydroxyoxan-2-yl)methyl 4-hydroxybenzoate | 0.0779 | up |
| metab_414 | Marmesin rhamnoside | 0.0722 | up |
| metab_64 | LysoPE(15:0/0:0) | 0.0700 | up |
| metab_4158 | Picolinic acid | 0.0682 | up |
| metab_1496 | D-Biotin | 0.0681 | up |
| metab_4258 | 1,4-Methylimidazoleacetic acid | 0.0623 | up |
| metab_3190 | Anandamide | 0.0616 | up |
| metab_3803 | Perindoprilat glucuronide | 0.0555 | up |
| metab_2758 | PE(17:0/0:0) | 0.0537 | up |
| metab_6516 | 19alpha-19-Hydroxy-3,11-dioxo-12-ursen-28-oic acid | 0.0510 | up |
| metab_4157 | Uracil | 0.0507 | up |
| metab_1486 | Myricanene B 5-[arabinosyl-(1->6)-glucoside] | 0.0450 | up |
| metab_990 | (25R)-3beta,4beta-dihydroxycholest-5-en-26-oate(1-) | 0.0440 | up |
| metab_3581 | 11-Dehydro-thromboxane B2 | 0.0318 | up |
| metab_1946 | Sphingosine | -0.0454 | down |
| metab_8227 | PE(18:0/0:0) | -0.0463 | down |
| metab_8260 | Polysorbate 20 | -0.0490 | down |
| metab_3256 | C16 Sphinganine | -0.0527 | down |
| metab_8224 | 13'-Carboxy-gamma-tocopherol | -0.0539 | down |
| metab_979 | Dihydroceramide | -0.0541 | down |
| metab_5748 | AH 6809 | -0.0565 | down |
| metab_140 | PC(16:0/0:0) | -0.0571 | down |
| metab_3570 | 12-hydroxyicosanoic acid | -0.0589 | down |
| metab_683 | 6-hydroxy-8-(2-hydroxy-3-methylbut-3-en-1-yl)-7-methoxy-2H-chromen-2-one | -0.0622 | down |
| metab_6735 | (3beta,5alpha,9alpha,22E,24R)-5,9-Epidioxy-3-hydroxyergosta-7,22-dien-6-one | -0.0658 | down |
| metab_3809 | Nuttalline | -0.0690 | down |
| metab_3067 | D-erythro-Sphingosine C-17 | -0.0717 | down |
| metab_6078 | LysoPE(0:0/24:6(6Z,9Z,12Z,15Z,18Z,21Z)) | -0.0726 | down |
| metab_2198 | 22,23-Dihydroergosterol | -0.0757 | down |
| metab_3014 | 2,3-dinor Prostaglandin E1 | -0.0838 | down |
| metab_2988 | Sphinganine | -0.0870 | down |
| metab_3841 | Undecanoic acid | -0.0919 | down |
| metab_3818 | Gingerenone C | -0.0962 | down |
| metab_3863 | Carboxyprimaquine | -0.1039 | down |
| metab_176 | Methyl 9,10-epoxy-12,15-octadecadienoate | -0.1115 | down |
| metab_3235 | Xestoaminol C | -0.1147 | down |
| metab_6717 | Tomentosic acid | -0.1151 | down |
| metab_2501 | Ustiloxin B | -0.1288 | down |
| metab_3649 | 2-Hydroxyacorenone | -0.1324 | down |
| metab_4237 | Pro Ser Ser | -0.1723 | down |
| metab_1601 | O-Desmethyltramadol | -0.1761 | down |
| metab_3814 | Thr Cys Glu Trp | -0.1869 | down |
| metab_9646 | 6-Hydroxyhexanoic acid | -0.2206 | down |
| metab_3847 | 13-Oxo-9,11-tridecadienoic acid | -0.2318 | down |
| metab_2880 | 10Z-Heptadecenoic acid | -0.2514 | down |
| metab_5226 | L-alpha-Hydroxyisovaleric acid | -0.2756 | down |
| metab_3039 | Avocadyne 4-acetate | -0.3731 | down |
| metab_4272 | Cytosine | -0.4456 | down |
| metab_8728 | SQDG(2:0/25:0) | -0.5688 | down |
| metab_1812 | 16-Hydroxy hexadecanoic acid | -0.5864 | down |
| metab_3716 | 2,4,4-Trimethylcyclopentanone | -0.6257 | down |
| metab_4159 | Methyl 3,4-dicaffeoylquinate | -1.4937 | down |
| metab_3295 | Solanocardinol | -1.5096 | down |
| metab_3743 | DL-2-Aminooctanoic acid | -1.7809 | down |
| metab_2860 | 4-[5]-ladderane-butanoic acid | -1.7844 | down |
