# Supplementary material for: Regulation of Sacha Inchi protein on fecal metabolism and intestinal microorganisms in mice
Source: Front Nutr. 2024 Mar 8;11:1354486. doi: 10.3389/fnut.2024.1354486 (PMC10959099; doi:10.3389/fnut.2024.1354486)
Supplement: Supplementary file 4 [file Table_3.DOCX]

Table S3 Significant metabolites differences between the SIP and WPI groups.

| Metab ID | Metabolite | Log_2_FC | Regulate |
| --- | --- | --- | --- |
| metab_2860 | 4-[5]-ladderane-butanoic acid | 2.1634 | up |
| metab_4120 | Tirofiban | 1.9760 | up |
| metab_8728 | SQDG(2:0/25:0) | 0.7480 | up |
| metab_5648 | (1alpha,2alpha,4betaH,6alpha,8R)-p-Menthane-2,6,8,9-tetrol | 0.7364 | up |
| metab_3716 | 2,4,4-Trimethylcyclopentanone | 0.6404 | up |
| metab_3698 | P-Acetaminobenzaldehyde | 0.5105 | up |
| metab_6563 | Ginsenoside Rh6 | 0.5046 | up |
| metab_3819 | Cucurbic acid | 0.3907 | up |
| metab_3039 | Avocadyne 4-acetate | 0.3664 | up |
| metab_1491 | 2-Naphthylamine | 0.3234 | up |
| metab_8638 | Arjunolic acid 3-glucoside | 0.2636 | up |
| metab_2880 | 10Z-Heptadecenoic acid | 0.2575 | up |
| metab_4237 | Pro Ser Ser | 0.2332 | up |
| metab_10015 | Gentisic acid | 0.2119 | up |
| metab_3818 | Gingerenone C | 0.2088 | up |
| metab_8134 | Sclareol | 0.2019 | up |
| metab_1108 | Leu Arg Met Leu Leu | 0.2006 | up |
| metab_797 | 2,6,10,10-Tetramethyl-1-oxaspiro[4.5]decan-6-ol | 0.1952 | up |
| metab_2501 | Ustiloxin B | 0.1940 | up |
| metab_1918 | 3-hydroxyhexanoyl carnitine | 0.1892 | up |
| metab_1883 | 15-cyclohexyl pentanor PGF2alpha | 0.1784 | up |
| metab_3649 | 2-Hydroxyacorenone | 0.1779 | up |
| metab_2092 | LysoPC(20:2(11Z,14Z)) | 0.1758 | up |
| metab_3168 | Isolinderanolide | 0.1670 | up |
| metab_3175 | Neuromedin N | 0.1609 | up |
| metab_2794 | Cis-7-Hexadecenoic Acid methyl ester | 0.1591 | up |
| metab_889 | Gln Lys Lys | 0.1568 | up |
| metab_4028 | P-Lactophenetide | 0.1565 | up |
| metab_3853 | L-1,2,3,4-Tetrahydro-beta-carboline-3-carboxylic acid | 0.1533 | up |
| metab_3010 | PS(14:1(9Z)/18:3(9Z,12Z,15Z)) | 0.1455 | up |
| metab_1601 | O-Desmethyltramadol | 0.1408 | up |
| metab_6717 | Tomentosic acid | 0.1399 | up |
| metab_1891 | 6-hydroxysphingosine | 0.1380 | up |
| metab_4756 | N-Acetyl-9-O-acetylneuraminic acid | 0.1353 | up |
| metab_9030 | Cibaric acid | 0.1304 | up |
| metab_176 | Methyl 9,10-epoxy-12,15-octadecadienoate | 0.1280 | up |
| metab_3841 | Undecanoic acid | 0.1243 | up |
| metab_3863 | Carboxyprimaquine | 0.1243 | up |
| metab_1100 | 13(S)-HODE methyl ester | 0.1222 | up |
| metab_6904 | 13,14-Dihydro-15-keto-PGE2 | 0.1153 | up |
| metab_138 | PC(18:1/0:0) | 0.1118 | up |
| metab_1029 | 11Z-hexadecenoic acid | 0.1102 | up |
| metab_1472 | Artabsin | 0.1092 | up |
| metab_3014 | 2,3-dinor Prostaglandin E1 | 0.1072 | up |
| metab_3054 | Germacrone-13-al | 0.1049 | up |
| metab_126 | PC(18:2/0:0) | 0.1019 | up |
| metab_2990 | 9,12-Octadecadiynoic Acid | 0.0965 | up |
| metab_3147 | 12-Hydroxy-8,10-octadecadienoic acid | 0.0959 | up |
| metab_6107 | Undecanedioic acid | 0.0955 | up |
| metab_3219 | (1S)-1-hydroxy-23-oxo-24,25,26,27-tetranorcalciol | 0.0945 | up |
| metab_3663 | 4-hydroxy-1-(4-methoxyphenyl)pentan-3-one | 0.0941 | up |
| metab_8316 | 11'-Carboxy-alpha-chromanol | 0.0933 | up |
| metab_6439 | (3beta,17alpha,23S)-17,23-Epoxy-3,29-dihydroxy-27-norlanost-8-en-24-one | 0.0885 | up |
| metab_122 | PA(12:0/22:6(4Z,7Z,10Z,13Z,16Z,19Z)) | 0.0872 | up |
| metab_1979 | Pinolenic Acid | 0.0854 | up |
| metab_985 | (+/-)9-HpODE | 0.0853 | up |
| metab_1101 | PC(P-16:0/0:0) | 0.0826 | up |
| metab_465 | Ile Val | 0.0823 | up |
| metab_9393 | Azelaic acid | 0.0807 | up |
| metab_3942 | 16-Hydroxy-10-oxohexadecanoic acid | 0.0798 | up |
| metab_2988 | Sphinganine | 0.0731 | up |
| metab_3390 | 3-[2-(3,7-dimethylocta-2,6-dien-1-yl)-3,4,6-trihydroxyphenyl]propanoic acid | 0.0703 | up |
| metab_585 | Eremopetasidione | 0.0696 | up |
| metab_140 | PC(16:0/0:0) | 0.0690 | up |
| metab_441 | Lotaustralin | 0.0650 | up |
| metab_8247 | 3beta,7alpha-Dihydroxy-5-cholestenoate | 0.0641 | up |
| metab_230 | 5-Methoxy-DL-tryptophan | 0.0634 | up |
| metab_6069 | 2,3-dinor, 6-keto-PGF1alpha | 0.0618 | up |
| metab_5965 | Sebacic acid | 0.0600 | up |
| metab_1013 | 9,10-DiHODE | 0.0558 | up |
| metab_866 | 13-epi-12-oxo Phytodienoic Acid | 0.0526 | up |
| metab_8224 | 13'-Carboxy-gamma-tocopherol | 0.0488 | up |
| metab_3500 | 12-OPDA | 0.0487 | up |
| metab_127 | 13(S)-HODE | 0.0484 | up |
| metab_707 | Corchorifatty acid D | 0.0454 | up |
| metab_3200 | postin | 0.0453 | up |
| metab_990 | (25R)-3beta,4beta-dihydroxycholest-5-en-26-oate(1-) | -0.0533 | down |
| metab_1335 | Descarbonyl-lacosamide | -0.0551 | down |
| metab_3803 | Perindoprilat glucuronide | -0.0569 | down |
| metab_3514 | D-Urobilin | -0.0587 | down |
| metab_6683 | PE(15:0/0:0) | -0.0630 | down |
| metab_3216 | Harderoporphyrinogen | -0.0666 | down |
| metab_6661 | PE(16:1/0:0) | -0.0667 | down |
| metab_6516 | 19alpha-19-Hydroxy-3,11-dioxo-12-ursen-28-oic acid | -0.0757 | down |
| metab_4954 | 2'-Deoxyuridine | -0.0767 | down |
| metab_3279 | Stearoylglycine | -0.0775 | down |
| metab_1007 | PE(14:0/0:0) | -0.0783 | down |
| metab_64 | LysoPE(15:0/0:0) | -0.0784 | down |
| metab_3190 | Anandamide | -0.0813 | down |
| metab_1709 | Enkephalin L | -0.0865 | down |
| metab_4819 | Lepidimoic acid | -0.0870 | down |
| metab_4435 | 7-Methylguanine | -0.0913 | down |
| metab_6098 | Mono-(2-ethyl-5-carboxypentyl) phthalate | -0.0948 | down |
| metab_4154 | Vidarabine | -0.1069 | down |
| metab_3920 | Kinetensin 4-7 | -0.1114 | down |
| metab_4888 | 7-Chloro-3,4',5,6,8-pentamethoxyflavone | -0.1125 | down |
| metab_10422 | 3,5,7-Trihydroxy-4',6-dimethoxyflavanone | -0.1151 | down |
| metab_1496 | D-Biotin | -0.1340 | down |
| metab_6509 | PE(13:0/0:0) | -0.1343 | down |
| metab_4152 | (5S,6S)-6-Amino-5-hydroxycyclohexane-1,3-diene-1-carboxyate | -0.1381 | down |
| metab_1297 | Obtusilactone A | -0.1562 | down |
| metab_7878 | 5-Phenylvaleric acid | -0.1586 | down |
| metab_4033 | Tragopogonsaponin G | -0.1644 | down |
| metab_3554 | (1(10)E,4a,5E)-1(10),5-Germacradiene-12-acetoxy-4,11-diol | -0.1701 | down |
| metab_1043 | 2-tetradecenal | -0.1780 | down |
| metab_1159 | Oleoyl Ethanolamide-d2 | -0.1790 | down |
| metab_849 | Tyr Arg Met Trp | -0.1828 | down |
| metab_4155 | 3-Methyl-5-propyl-2-furanundecanoic acid | -0.2106 | down |
| metab_3234 | Dihomo-gamma-Linolenoyl ethanolamide | -0.2408 | down |
| metab_3252 | Temazepam | -0.2676 | down |
| metab_1384 | 5-Aminopentanal | -0.2893 | down |
| metab_3712 | Methyl 5-hydroxyoxindole-3-acetate | -0.2999 | down |
| metab_4111 | Lacto-N-triose I | -0.3189 | down |
| metab_5201 | (+/-)-2-Hydroxy-4-(methylthio)butanoic acid | -0.3228 | down |
| metab_3654 | 2-Hydroxyphenylacetic acid | -0.3263 | down |
| metab_1322 | 9-Chloro-17beta-hydroxy-17-methylandrost-4-ene-3,11-dione | -0.3395 | down |
| metab_3970 | Cyclolinopeptide F | -0.4695 | down |
| metab_8301 | PS(14:1(9Z)/20:5(5Z,8Z,11Z,14Z,17Z)) | -0.5406 | down |
| metab_488 | Grepafloxacin | -0.6427 | down |
| metab_4108 | Kanzonol O | -0.7403 | down |
| metab_3690 | (E)-10-Hydroxy-2-decene-4,6-diynoic acid | -0.7466 | down |
| metab_7033 | 6-pentadecyl Salicylic Acid | -0.7779 | down |
| metab_4843 | 3'-(6''-Galloylglucosyl)-phloroacetophenone | -0.7876 | down |
| metab_4617 | Taurine | -0.8357 | down |
| metab_1008 | 2-tridecenal | -0.8468 | down |
| metab_6096 | Mono-(2-ethyl-5-oxohexyl) phthalate | -0.9526 | down |
| metab_2164 | Ganolucidic acid B | -0.9675 | down |
| metab_3859 | Valyl-Valine | -0.9834 | down |
| metab_3990 | Withaperuvin B | -1.1546 | down |
| metab_4309 | 1-[(5-Amino-5-carboxypentyl)amino]-1-deoxyfructose | -2.5096 | down |
| metab_1339 | 16-iodo-hexadecanoic acid | -2.7573 | down |
